# Interaction between body mass index and hormone-receptor status as a prognostic factor in lymph-node-positive breast cancer

**DOI:** 10.1371/journal.pone.0170311

**Published:** 2017-03-01

**Authors:** Il Yong Chung, Jong Won Lee, Ji Sung Lee, Yu Rang Park, Yul Ha Min, Yura Lee, Tae In Yoon, Guiyun Sohn, Sae Byul Lee, Jisun Kim, Hee Jeong Kim, Beom Seok Ko, Byung Ho Son, Sei Hyun Ahn

**Affiliations:** 1 Department of Surgery, University of Ulsan College of Medicine, Asan Medical Center, Seoul, Republic of Korea; 2 Clinical Research Center, University of Ulsan College of Medicine, Asan Medical Center, Seoul, Republic of Korea; 3 Gachon University College of Nursing, Incheon, Republic of Korea; University of Cincinnati College of Medicine, UNITED STATES

## Abstract

The aim of this study was to determine the relationship between the body mass index (BMI) at a breast cancer diagnosis and various factors including the hormone-receptor, menopause, and lymph-node status, and identify if there is a specific patient subgroup for which the BMI has an effect on the breast cancer prognosis. We retrospectively analyzed the data of 8,742 patients with non-metastatic invasive breast cancer from the research database of Asan Medical Center. The overall survival (OS) and breast-cancer-specific survival (BCSS) outcomes were compared among BMI groups using the Kaplan-Meier method and Cox proportional-hazards regression models with an interaction term. There was a significant interaction between BMI and hormone-receptor status for the OS (*P* = 0.029), and BCSS (*P* = 0.013) in lymph-node-positive breast cancers. Obesity in hormone-receptor-positive breast cancer showed a poorer OS (adjusted hazard ratio [HR] = 1.51, 95% confidence interval [CI] = 0.92 to 2.48) and significantly poorer BCSS (HR = 1.80, 95% CI = 1.08 to 2.99). In contrast, a high BMI in hormone-receptor-negative breast cancer revealed a better OS (HR = 0.44, 95% CI = 0.16 to 1.19) and BCSS (HR = 0.53, 95% CI = 0.19 to 1.44). Being underweight (BMI < 18.50 kg/m^2^) with hormone-receptor-negative breast cancer was associated with a significantly worse OS (HR = 1.98, 95% CI = 1.00–3.95) and BCSS (HR = 2.24, 95% CI = 1.12–4.47). There was no significant interaction found between the BMI and hormone-receptor status in the lymph-node-negative setting, and BMI did not interact with the menopause status in any subgroup. In conclusion, BMI interacts with the hormone-receptor status in a lymph-node-positive setting, thereby playing a role in the prognosis of breast cancer.

## Introduction

There has been some controversy about whether the body mass index (BMI) at the diagnosis of breast cancer is associated with the patient outcome. Several clinical trials have demonstrated that obesity at diagnosis is associated with breast-cancer-specific survival (BCSS) and overall survival (OS) [1–3], which was additionally supported by recent meta-analyses [4]. However, other studies have found no association between BMI and the prognosis of breast cancer [5–7].

Considering the heterogeneity of breast cancer, it is possible that the effect of BMI on its prognosis is influenced by various factors [3]. Many studies have been conducted to find such specific factors, with the breast cancer subtype being one of the most widely investigated, but the results have been inconsistent. Some studies reported that obesity is a prognostic factor in hormone-receptor-positive breast cancer. [8, 9] In contrast, a previous meta-analysis reported that the association of obesity with breast cancer outcome does not differ by hormone receptor [6], and another study of data from four separate clinical trials reported no consistent relationship between BMI at diagnosis and breast cancer death or recurrence [3]. A further study which analyzed the data from a clinical trial could find no interaction between BMI and molecular subtype in terms of breast cancer outcomes [8, 10].

There is also some controversy as to the impact of menopause which is another factor that has been studied in this context. Investigators from the EBCTCG (Early Breast Cancer Trialists’ Collaborative Group) reported that obesity is a negative prognostic indicator in premenopausal patients with estrogen-receptor-positive breast cancer, while others have found that obesity was an adverse prognostic factor in postmenopausal breast cancer patients [8, 10]. In contrast, a previous meta-analysis of 82 studies concluded that obesity is associated with a poorer survival outcome regardless of the menopause status [11]. Recently, the lymph node status has been suggested for consideration as a specific factor in analyzing the effect of BMI on breast cancer prognosis. The previous analysis of four separate clinical trials revealed that BMI had a prognostic role in estrogen-receptor-positive breast cancer patients only in the two trials that enrolled lymph-node-positive breast cancer patients, but that these results were different in another trial with node-positive settings [3].

It is likely that the complexity of the relationships between the BMI and various known factors is responsible for its varying reported effects on breast cancer survival between studies. Moreover, most of the previous studies investigating the association between BMI and other factors did not include explanatory variables such as tumor grade, lymphovascular invasion and treatment, and the definition of menopausal status has differed between trials [3, 12]. Furthermore, most previous studies have focused on subgroup analyses and did not demonstrate interactions between BMI and various factors, with few if any investigating interactions between BMI and other factors from the aspect of breast cancer outcomes [3, 12]. In our current study, we hypothesized that BMI has an effect on breast cancer outcome in a specific subgroup which could be investigated using subgroup analysis according to lymph-node, hormone-receptor and menopause status using more comprehensive explanatory variables. We performed analyses with interaction terms to obtain a better understanding of the relationship between BMI and these subgroups of breast cancer patients.

The aim of this study therefore was to determine the relationship between the BMI at diagnosis and known factors including the hormone-receptor and menopause status of the patients, and to identify if there is a specific breast cancer population according to lymph-node status for which BMI has an effect on the prognosis.

## Materials and methods

### Patients

Data on breast cancer patients treated at our institution between January 1997 and June 2008 were retrieved from the Asan Medical Center research database, a web-based system that includes archived anonymous information on all patients who undergo breast cancer surgery. Patients were included in the study if they were aged 80 years or younger at the time of diagnosis, were diagnosed with stage I to stage III breast cancer, and were treated surgically. The patients for whom information on the BMI at diagnosis was not available or who received neoadjuvant systemic therapy were excluded. In total, 8,742 patients were finally included in the analysis. This study was approved by the Institutional Review Board of Asan Medical Center (Approval No. 2015–0924).

### Measurements

The following information was available on the study patients: age, weight, height, tumor size, number of metastatic lymph nodes, estrogen-receptor status, progesterone-receptor status, histology grade, lymphovascular invasion, menopause status, and the administration of chemotherapy, radiation therapy, and hormonal therapy. The weight and height of each patient were measured during the hospital stay prior to breast cancer surgery. The BMI was calculated from these measurements and categorized according to the International Classification of the World Health Organization i.e. underweight (UW, BMI < 18.5 kg/m2), normal weight (NW, BMI = 18.5–24.9 kg/m2), overweight (OW, BMI = 25–29.9 kg/m2) and obese (OB, BMI ≥ 30 kg/m2).

### Statistical analysis

Descriptive statistics were summarized with absolute and relative frequencies. Continuous and categorical variables were compared among BMI groups using one-way analysis of variance and the χ2 test, respectively. The breast cancer outcome was analyzed in terms of OS and BCSS, defined as the time from the first diagnosis of primary breast cancer to death from any cause and death from breast cancer, respectively. The survival of patients who were lost to follow-up was calculated using the last date of follow-up. The survival curves for all four BMI groups were estimated using the Kaplan-Meier method, and the log-rank test was used to evaluate statistical significance. To assess the BMI category as a prognostic factor for OS and BCSS, multivariate analyses were conducted using Cox proportional-hazards regression models adjusted for previously known prognostic factors, including age, tumor size, the number of pathologically confirmed positive lymph nodes, histology grade (low or high), hormone-receptor status (positive or negative), treatment (radiation therapy, chemotherapy) and menopause status (premenopausal or postmenopausal), with NW as a reference category. Estrogen receptor and/or progesterone receptor positivity was defined as a positive hormone-receptor status. Interaction effects between BMI and other factors (hormone-receptor or menopause status) were explored by adding interaction terms to the model in which BMI was treated as an ordinal. The patients were divided into two subgroups according to their lymph-node status (positive or negative), and subgroup analyses were performed to evaluate whether the effect of BMI on breast cancer outcome differed in different settings.

Statistical analyses were performed using R software (version 3.2.4, R Foundation for Statistical Computing, Vienna, Austria) and SAS software (version 9.4, SAS Institute, NC). A two-sided *P* value of <0.05 was considered statistically significant.

## Results

### Patients

The patients were aged 47.7± 9.9 years (mean ± SD, range = 19–80 years) with a mean BMI of 23.6 ± 3.2 kg/m2 (range = 12.6–46.8 kg/m2). Table 1 lists the clinicopathologic characteristics of the different BMI groups. The OB patients were the oldest, and there were some noted differences in tumor size, tumor stage, axillary lymph-node metastasis, histology grade, and chemotherapy between BMI groups.

**Table 1:** Clinicopathologic characteristics of the different body mass index groups.

	UW (*n* = 247)		NW (*n* = 6,009)		OW (*n* = 2,165)		OB (*n* = 320)		
Characteristic	*n*	%	*n*	%	*n*	%	*n*	%	*p*
Age, years (mean±SD)	41.3±9.9		46.3±9.2		51.3±10.2		53.5±10.9		<0.001
Tumor size, cm (mean±SD)	1.96±1.63		2.29±1.78		2.54±1.75		2.64±1.73		<0.001
Tumor stage									
T1	167	67.9	3,407	56.9	1,020	47.3	123	38.4	<0.001
>T1	79	32.1	2,583	43.1	1,138	52.7	197	61.6	
Axillary lymph-node metastasis									
Negative	160	65.3	3,616	60.2	1,207	55.9	184	57.5	<0.001
Positive	85	34.7	2,386	39.8	953	44.1	136	42.5	
Hormone-receptor status									
Negative	86	35.7	2,036	34.2	771	35.9	115	36.3	0.512
Positive	155	64.3	3,911	65.8	1,378	64.1	202	63.7	
Histology grade									
Low (1 or 2)	139	65	3,355	61.7	1,171	58.9	165	57.3	0.046
High (3)	75	35	2,081	38.3	816	41.1	123	42.7	
Lymphovascular invasion									
Absent	140	73.7	3,380	72.7	1,197	72.5	176	69.8	0.719
Present	50	26.3	1,269	27.3	455	27.5	76	30.2	
Radiation therapy									
No	100	41.2	2,513	42	922	42.7	140	44	0.832
Yes	143	58.8	3,473	58	1,238	57.3	178	56	
Hormonal therapy									
No	88	36.7	1,800	30.3	652	30.6	108	34.1	0.102
Yes	152	63.3	4,142	69.7	1,482	69.4	209	65.9	
Chemotherapy									
No	95	40.1	1,801	30.6	560	26.5	88	27.8	<0.001
Yes	142	59.9	4,113	69.4	1,556	73.5	228	72.2	

UW, underweight; NW, normal weight; OW, overweight; OB, obese; SD, standard deviation.

### Survival analysis of the total population and body mass index subgroups according to lymph-node status

**Fig 1:**
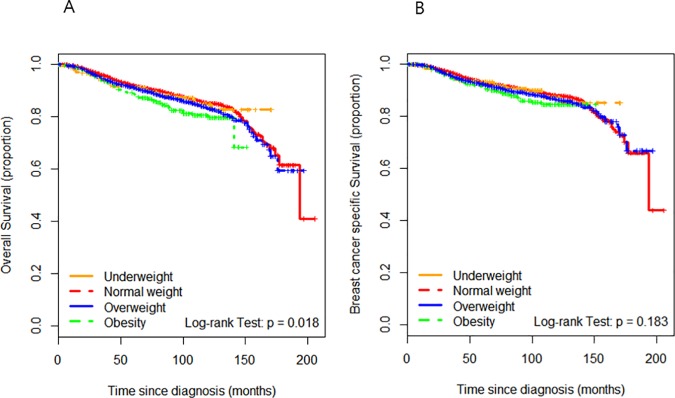
Overall survival and breast-cancer-specific survival curves according to the four body mass index groups. (A) Overall survival. (B) Breast-cancer-specific survival.

During a median follow-up of 92 months, there were 1,178 deaths from any cause in our study cohort and 957 patients died of breast cancer. Ninety-eight patients died of other causes which included other cancers in 65 cases, trauma in 8 cases, cardiovascular disease in 6 cases, chronic liver disease in 5 cases, sepsis in 3 cases, acute respiratory distress syndrome in 2 cases, cerebrovascular disease in 2 cases, autoimmune disease in 2 cases, suicide in 1 case and other medical problems in 4 cases. The cause of mortality was unknown in 123 patients. Among the total study population, univariate analysis revealed significant differences in OS outcomes between BMI groups but not in terms of BCSS (Fig 1). However, by multivariate analysis, there were no significant intergroup differences in the OS (LW, *P* = 0.079; OW, *P* = 0.725; OB, *P* = 0.238) or BCSS (LW, *P* = 0.219; OW, *P* = 0.330; OB, *P* = 0.186). Among the lymph-node-positive breast cancer patients, neither univariate nor multivariate analysis revealed any significant difference between the BMI groups in OS (LW, *P* = 0.261; OW, *P* = 0.721; OB, *P* = 0.834) or BCSS (LW, *P* = 0.210; OW, *P* = 0.391; OB, *P* = 0.352) outcomes. Moreover, in lymph-node-negative settings, there was no significant intergroup difference found in the OS (LW, *P* = 0.316; OW, *P* = 0.762; OB, *P* = 0.225) or BCSS (LW, *P* = 0.944; OW, *P* = 0.937; OB, *P* = 0.545).

### Survival analysis in body mass index subgroups according to hormone-receptor or menopause status

**Fig 2:**
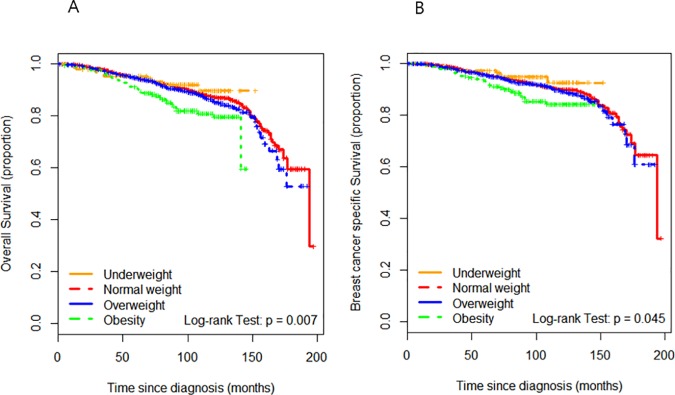
Overall survival and breast-cancer-specific survival curves for the hormone-receptor-positive breast cancer patients according to the four body mass index groups. (A) Overall survival. (B) Breast-cancer-specific survival.

Among the hormone-receptor-positive breast cancer patients in our study cohort (n = 4,363), univariate analysis indicated that obesity was a negative prognostic indicator for both OS and BCSS (Fig 2) and multivariate analysis revealed that the OB group exhibited a trend toward a poorer OS (adjusted hazard ratio [HR] = 1.48, 95% confidence interval [CI] = 0.97–2.25) and a significantly poorer BCSS (HR = 1.65, 95% CI = 1.02–2.66). Among the hormone-receptor-negative breast cancer cases (n = 1,859), the OS and BCSS did not differ significantly among BMI groups by univariate analysis. However, by multivariate analysis we found that the patients in the UW group exhibited a poorer OS (HR = 1.72, 95% CI = 0.97–3.05) and significantly poorer BCSS (HR = 1.90, 95% CI = 1.05–3.46). Subgroup analyses further revealed that in the premenopausal patients (n = 4,321), obesity was associated with a significantly poorer OS (HR = 1.87, 95% CI = 1.20 to 2.91) and BCSS (HR = 2.04, 95% CI = 1.27 to 3.26) by multivariate analysis.

### Interaction between BMI and hormone-receptor or menopause status

**Fig 3:**
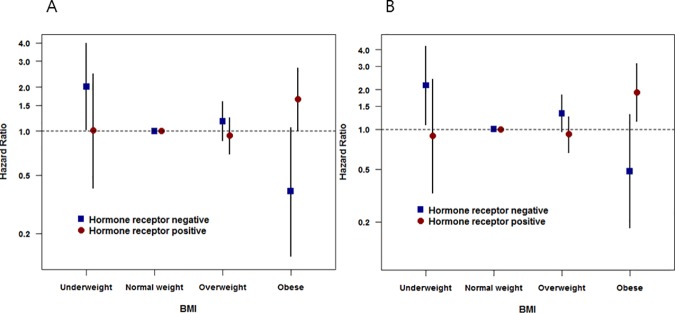
Interaction between body mass index and hormone-receptor status for overall survival and breast-cancer-specific survival in lymph-node-positive breast cancer patients. (A) Overall survival. (B) Breast-cancer-specific survival. Error bars show 95% confidence intervals.

In the total study population, a significant interaction was found between BMI and the hormone-receptor status for the BCSS outcome (*P* = 0.039), but not for OS (*P* = 0.0623). However, multivariate analysis demonstrated a significant interaction between BMI and hormone-receptor status in terms of both the OS (*P* = 0.029) and BCSS (*P* = 0.013) in lymph-node-positive breast cancers. Obesity in hormone-receptor-positive breast cancer cases was associated with a poorer OS (HR = 1.51, 95% CI = 0.92 to 2.48) and significantly poorer BCSS (HR = 1.80, 95% CI = 1.08 to 2.99), but a high BMI in hormone-receptor-negative breast cancer correlated with both a better OS (HR = 0.44, 95% CI = 0.16 to 1.19) and BCSS (HR = 0.53, 95% CI = 0.19 to 1.44). In contrast, UW in hormone-receptor-negative breast cancer demonstrated a trend toward a worse OS and significantly worse BCSS (Table 2). The effect of BMI on breast cancer prognosis differed with the hormone receptor status in the lymph-node-positive setting (Fig 3). However, among the lymph-node-negative breast cancer patients, there was no significant interaction found (OS, *P* = 0.874; BCSS, *P* = 0.738) between BMI and hormone-receptor status (Table 3). There was also no significant interaction observed between BMI and menopause status in terms of the OS (lymph-node positive, *P* = 0.088; lymph-node negative, *P* = 0.466) or BCSS (lymph-node positive, *P* = 0.055; lymph-node negative, *P* = 0.387) in any subgroup (Tables A and B in S1 File).

**Table 2:** Cox proportional-hazards regression models with an interaction term for body mass index and hormone-receptor status in lymph-node-positive breast cancer patients.

		OS		BCSS	
Characteristic	N	HR (95% CI)	*p*	HR (95% CI)	*p*
Age at diagnosis		0.99 (0.98–1.00)	0.172	0.99 (0.97–1.00)	0.0805
Tumor size		1.10 (1.08–1.13)	<0.001	1.11 (1.08–1.13)	<0.001
Number of positive lymph nodes		1.05 (1.04–1.06)	<0.001	1.05 (1.04–1.06)	<0.001
Histology grade (low)		0.55 (0.45–0.68)	<0.001	0.53 (0.42–0.66)	<0.001
Lymphovascular invasion		1.52 (1.13–2.05)	<0.001	1.54 (1.23–1.92)	<0.001
Postmenopausal		1.52 (1.13–2.05)	0.006	1.49 (1.08–2.06)	0.015
Radiation therapy (performed)		0.70 (0.56–0.88)	0.002	0.69 (0.54–0.87)	0.002
Chemotherapy (performed)		0.38 (0.24–0.60)	<0.001	0.53 (0.30–0.93)	0.028
BMI (hormone-receptor negativity)					
NW	481	1.00	-	1.00	-
UW	20	1.98 (1.00–3.95)	0.051	2.24 (1.12–4.47)	0.023
OW	177	1.22 (0.88–1.68)	0.229	1.36 (0.98–1.91)	0.069
OB	26	0.44 (0.16–1.19)	0.105	0.53 (0.19–1.44)	0.213
BMI (hormone-receptor positivity)					
NW	1,203	1.00	-	1.00	-
UW	43	0.90 (0.37–2.19)	0.812	0.82 (0.30–2.23)	0.697
OW	476	0.92 (0.69–1.22)	0.548	0.92 (0.67–1.26)	0.606
OB	75	1.51 (0.92–2.48)	0.100	1.80 (1.08–2.99)	0.024

UW, underweight; NW, normal weight; OW, overweight; OB, obese; HR, adjusted hazard ratio; CI, confidence interval.

*p* = 0.029 for interaction effect between body mass index and hormone-receptor status in overall survival.

*p* = 0.013 for interaction effect between body mass index and hormone receptor status in breast-cancer-specific survival.

**Table 3:** Cox proportional-hazards regression models with an interaction term for body mass index and hormone-receptor status in lymph-node-negative breast cancer patients.

		OS		BCSS	
Characteristic	N	HR (95% CI)	*p*	HR (95% CI)	*p*
Age at diagnosis		1.02 (1.00–1.04)	0.017	1.00 (0.98–1.03)	0.741
Tumor size		1.19 (1.11–1.28)	<0.001	1.21 (1.12–1.31)	<0.001
Histology grade (low)		0.66 (0.49–0.89)	0.006	0.53 (0.37–0.75)	<0.001
Lymphovascular invasion		1.68 (1.21–2.32)	0.002	1.98 (1.36–2.89)	<0.001
Postmenopausal		1.16 (0.79–1.72)	0.455	1.29 (0.80–2.08)	0.302
Radiation therapy (performed)		0.90 (0.70–1.17)	0.447	1.08 (0.78–1.50)	0.626
Chemotherapy (performed)		1.24 (0.87–1.78)	0.238	1.81 (1.12–2.95)	0.017
BMI (hormone-receptor negativity)					
NW	786	1.00	-	1.00	-
UW	37	1.43 (0.52–3.94)	0.486	1.19 (0.37–3.81)	0.776
OW	280	1.08 (0.70–1.66)	0.721	1.12 (0.69–1.81)	0.651
OB	52	1.49 (0.74–3.01)	0.262	1.55 (0.70–3.43)	0.275
BMI (hormone-receptor positivity)					
NW	1,832	1.00	-	1.00	-
UW	72	1.46 (0.54–4.00)	0.458	0.64 (0.09–4.61)	0.654
OW	582	0.84 (0.55–1.30)	0.441	0.89 (0.50–1.58)	0.696
OB	80	1.28 (0.58–2.79)	0.541	0.74 (0.18–3.05)	0.673

UW, underweight; NW, normal weight; OW, overweight; OB, obese; HR, adjusted hazard ratio; CI, confidence interval.

*p* = 0.874 for interaction effect between body mass index and hormone-receptor status in overall survival.

*p* = 0.738 for interaction effect between body mass index and hormone-receptor status in breast-cancer-specific survival.

## Discussion

The findings of our current study indicate that an interaction between the BMI and the hormone-receptor status at the diagnosis of breast cancer plays a role as a prognostic factor for this disease only in the lymph-node-positive setting. Obesity was found to be associated with significantly poorer breast-cancer-specific survival in hormone-receptor-positive breast cancer, but a better survival outcome in hormone-receptor-negative breast cancer cases, although this was not statistically significant. However, UW in hormone-receptor-negative breast cancer demonstrated a trend toward a poorer OS and significantly poorer BCSS. There was no significant interaction found between BMI and hormone receptor in lymph-node-negative breast cancer patients. Additionally, there was no significant interaction found between BMI and menopause status in any of the study populations.

To our knowledge, this is the first study to demonstrate an interaction between BMI and the hormone-receptor status on breast cancer prognosis. Most of the previous studies that investigated this relationship used subgroup analyses. However, only two previous studies have investigated the interaction between BMI and breast cancer subtype on the clinical outcome, and these reports could not demonstrate any statistically significant interaction between these two factors [3, 12]; however, this may have been due to the small sample size. The authors from the Cancer and Leukemia Group 9741 previously analyzed about 1,300 patients, and researchers in a separate analysis who compared the data from four different randomized controlled trials included about 3,500 patients. In our present study, we were able to conduct interaction tests with much larger cohorts, which may have improved our ability to detect statistically significant differences.

Our present study is one of the few to suggest that in specific cases of lymph-node-positive breast cancer, BMI has an effect on the prognosis. We are aware of only one other study that has suggested obesity to be associated with poorer survival outcomes in two trials which enrolled lymph-node-positive breast cancer patients [3]. However, that previous study did not show a consistent relationship between BMI and breast cancer prognosis in another trial with a lymph-node-positive setting. These inconsistencies may have been due to differences in the data sets. In our current retrospective cohort study, additional explanatory variables including tumor grade, lymphovascular invasion and treatment were available for analysis.

There has been some controversy to date concerning the relationship between BMI and menopause status in the prognosis of breast cancer [8, 10, 11]. In our present study, although subgroup analyses by menopause status indicated that obesity was a significant prognostic factor in the premenopausal subgroup for both OS (HR = 1.87, 95% CI = 1.20–2.91) and BCSS (HR = 2.04, 95% CI = 1.28–3.26), the subsequent interaction test revealed no significant interaction between BMI and menopause status in terms of the breast cancer prognosis in any of our subgroups (Tables A and B in S1 File). However, in our lymph-node-positive subgroup, we observed a trend toward an interaction between BMI and menopause status for the BCSS (*P* = 0.055), and obesity showed an association with a poorer prognosis in premenopausal women (HR = 1.94, 95% CI = 1.14–3.29). Additionally, we explored the correlation between hormone-receptor status and menopause status using the χ2 test which revealed a significant association between these factors in our study population (*P* = 0.006). The rate of hormone-receptor positive breast cancers was higher for patients with a premenopausal status (72%, 4,242/5,931) compared with a postmenopausal status (64%, 1,643/2,571), which could influence the association between BMI and cancer outcome. Future studies with greater statistical power will likely be useful for determining the interaction between BMI and menopause status in the prognosis of breast cancer.

In terms of using BMI as a prognostic factor in breast cancer, our current results suggest the importance of the lymph-node status. Several reports have indicated that BMI is a prognostic indicator in locally advanced breast cancer [8, 13]. The influence of lymph-node positivity on BMI is not fully understood [3]. Further research is thus needed to obtain a better understanding of this finding.

Several earlier studies have reported that not only obesity but also UW is a negative prognostic indicator in breast cancer [9, 14–16]. Some authors have recently demonstrated a relationship between UW and breast cancer subtype with respect to outcome. They reported that UW was associated with both a poorer OS (HR = 1.68, 95% CI = 1.12–2.47) and BCSS (HR = 1.79, 95% CI = 1.11–2.90), but only in hormone receptor positive and human epidermal growth factor receptor 2 negative breast cancer. Likewise, we here found a significant interaction between UW and a negative hormone-receptor status for a poor prognosis only in lymph-node-positive disease, although UW was not found to be associated with worse prognosis in our total study population or in the lymph-node-negative setting.

There are several possible hypotheses for explaining the biological mechanisms connecting the BMI to a worse prognosis in breast cancer patients [17]. Obesity is positively correlated with an elevated level of circulating estrogens and inversely correlated with the plasma sex hormone-binding globulins, both of which can promote the progression of hormone-receptor-positive breast cancers. Hyperinsulinemia caused by obesity can lead to resistance to endocrine therapy and breast cancer recurrence [17, 18]. Malnutrition due to UW may compromise immune function and surveillance, thereby playing a role in the prognosis of breast cancer in the hormone-receptor-negative setting, which tends to be associated with a more aggressive type of tumor [19]. Additionally, the failure to complete adjuvant chemotherapy seems to occur more frequently in UW patients and is therefore a possible explanation for the poorer prognosis in hormone-receptor-negative breast cancer cases.

Several limitations of our present study should be noted. This was a retrospective and single-institutional study that may have been influenced by selection bias. Although we performed multivariate analysis with known prognostic factors, we were not able to include other significant prognostic factors such as the expression of human epidermal growth factor receptor 2, which may influence the association between BMI and cancer outcome. In addition, although our sample size was larger than that of previous studies, the number of UW and OB patients in our cohort was relatively small, which could have affected the statistical power of our subgroup analysis. Moreover, some of our survival data were obtained from the Korean national insurance database which gives information on the date of death but not the cause. The number of deaths of unknown cause among our study population was therefore relatively high. Moreover, our study population mainly comprised Korean patients, and our findings may therefore only be generalizable to Asian populations.

In conclusion, our current study has yielded clues regarding the complex relationships between BMI and various factors affecting breast cancer outcomes. The BMI interacts with hormone-receptor status in the lymph-node-positive setting, thereby playing a role in the prognosis of breast cancer. Our findings support the application of an individualized approach to the management of breast cancer survivors, given that the different role of the BMI at diagnosis differs depending on the hormone-receptor status in relation to the lymph-node status.

## Supporting information

S1 File Table A. Cox proportional-hazards regression models with an interaction term for body mass index and menopause status in lymph-node-positive breast cancer patients. Table B. Cox proportional-hazards regression models with an interaction term for body mass index and menopause status in lymph-node-negative breast cancer patients.
(DOCX)

S1 Dataset (XLSX)
