# Supplementary material for: Interaction between body mass index and hormone-receptor status as a prognostic factor in lymph-node-positive breast cancer
Source: PLoS One. 2017 Mar 1;12(3):e0170311. doi: 10.1371/journal.pone.0170311 (PMC5331962; doi:10.1371/journal.pone.0170311)
Supplement: S1 File — Table A. Cox proportional-hazards regression models with an interaction term for body mass index and menopause status in lymph-node-positive breast cancer patients. Table B. Cox proportional-hazards regression models with an interaction term for body mass index and menopause status in lymph-node-negative breast cancer patients. (DOCX) [file pone.0170311.s001.docx]

**Table A. Cox proportional-hazards regression models with an interaction term for body mass index and menopause status in lymph-node-positive breast cancer patients.**

|  | OS | |  | BCSS | |
| --- | --- | --- | --- | --- | --- |
| Characteristic | HR (95% CI) | *p* |  | HR (95% CI) | *p* |
| Age at diagnosis | 0.99 (0.98–1.00) | 0.136 |  | 0.99 (0.97–1.00) | 0.0569 |
| Tumor size | 1.10 (1.07–1.12) | <0.001 |  | 1.11 (1.08–1.13) | <0.001 |
| Number of positive lymph nodes | 1.05 (1.04–1.06) | <0.001 |  | 1.05 (1.04–1.06) | <0.001 |
| Histology grade (low) | 0.54 (0.44–0.67) | <0.001 |  | 0.52 (0.41–0.65) | <0.001 |
| Lymphovascular invasion | 1.55 (1.26–1.90) | <0.001 |  | 1.57 (1.25–1.96) | <0.001 |
| Hormone-receptor positivity | 0.63 (0.51–0.77) | <0.001 |  | 0.61 (0.49–0.76) | <0.001 |
| Radiation therapy (performed) | 0.70 (0.56–0.87) | 0.001 |  | 0.69 (0.54–0.87) | 0.002 |
| Chemotherapy (performed) | 0.35 (0.22–0.56) | <0.001 |  | 0.48 (0.27–0.85) | 0.012 |
| BMI (postmenopausal) |  |  |  |  |  |
| NW | 1.00 | - |  | 1.00 | - |
| UW | 3.02 (0.73–12.42) | 0.126 |  | 4.09 (0.99–16.94) | 0.052 |
| OW | 1.03 (0.74–1.43) | 0.881 |  | 1.16 (0.80–1.67) | 0.432 |
| OB | 0.57 (0.26–1.24) | 0.155 |  | 0.65 (0.28–1.49) | 0.306 |
| BMI (premenopausal) |  |  |  |  |  |
| NW | 1.00 | - |  | 1.00 | - |
| UW | 1.26 (0.70–2.27) | 0.442 |  | 1.28 (0.69–2.38) | 0.430 |
| OW | 1.03 (0.78–1.37) | 0.835 |  | 1.05 (0.78–1.42) | 0.735 |
| OB | 1.66 (0.98–2.81) | 0.059 |  | 1.94 (1.14–3.29) | 0.014 |

UW, underweight; NW, normal weight; OW, overweight; OB, obese; HR, adjusted hazard ratio; CI, confidence interval.

*p* = 0.0881 for interaction effect between body mass index and menopause status in overall survival.

*p* = 0.0554 for interaction effect between body mass index and menopause status in breast-cancer-specific survival.

**Table B. Cox proportional-hazards regression models with an interaction term for body mass index and menopause status in lymph-node-negative breast cancer patients.**

|  | OS | |  | BCSS | |
| --- | --- | --- | --- | --- | --- |
| Characteristic | HR (95% CI) | *p* |  | HR (95% CI) | *p* |
| Age at diagnosis | 1.02 (1.00–1.04) | 0.017 |  | 1.00 (0.98–1.03) | 0.796 |
| Tumor size | 1.19 (1.11–1.28) | <0.001 |  | 1.21 (1.12–1.31) | <0.001 |
| Histology grade (low) | 0.66 (0.49–0.88) | 0.005 |  | 0.52 (0.36–0.74) | <0.001 |
| Lymphovascular invasion | 1.67 (1.20–2.31) | 0.002 |  | 1.98 (1.36–2.88) | <0.001 |
| Hormone-receptor positivity | 0.69 (0.50–0.95) | 0.024 |  | 0.62 (0.42–0.91) | 0.014 |
| Radiation therapy (performed) | 0.90 (0.69–1.17) | 0.445 |  | 1.09 (0.78–1.50) | 0.603 |
| Chemotherapy (performed) | 1.23 (0.86–1.77) | 0.256 |  | 1.80 (1.11–2.93) | 0.018 |
| BMI (postmenopausal) |  |  |  |  |  |
| NW | 1.00 | - |  | 1.00 | - |
| UW | 1.55 (0.48–4.98) | 0.461 |  | 1.66 (0.40–6.99) | 0.489 |
| OW | 1.14 (0.75–1.73) | 0.550 |  | 1.34 (0.78–2.28) | 0.286 |
| OB | 1.24 (0.63–2.44) | 0.538 |  | 1.13 (0.44–2.91) | 0.798 |
| BMI (premenopausal) |  |  |  |  |  |
| NW | 1.00 | - |  | 1.00 | - |
| UW | 1.46 (0.56–3.42) | 0.486 |  | 0.67 (0.16–2.76) | 0.582 |
| OW | 0.76 (0.48–1.23) | 0.265 |  | 0.78 (0.45–1.35) | 0.378 |
| OB | 1.90 (0.83–4.35) | 0.127 |  | 1.61 (0.58–4.41) | 0.359 |

UW, underweight; NW, normal weight; OW, overweight; OB, obese; HR, adjusted hazard ratio; CI, confidence interval.

*p* = 0.466 for interaction effect between body mass index and menopause status in overall survival.

*p* = 0.388 for interaction effect between body mass index and menopause status in breast-cancer-specific survival.
